# Blood-Based Kinase Assessments in Alzheimer’s Disease

**DOI:** 10.3389/fnagi.2018.00338

**Published:** 2018-11-14

**Authors:** Jacques Hugon, François Mouton-Liger, Emmanuel Cognat, Julien Dumurgier, Claire Paquet

**Affiliations:** ^1^Center of Cognitive Neurology, Lariboisiere Fernand-Widal Hospital, APHP, University Paris Diderot, Paris, France; ^2^INSERM U 942, Paris, France

**Keywords:** Alzheimer, kinase, blood, biomarkers, diagnosis

## Abstract

Alzheimer’s disease (AD) is marked by memory disturbances followed by aphasia, apraxia and agnosia. Brain lesions include the accumulation of the amyloid peptide in extracellular plaques, neurofibrillary tangles with abnormally phosphorylated tau protein and synaptic and neuronal loss. New findings have suggested that brain lesions could occur one or two decades before the first clinical signs. This asymptomatic preclinical phase could be an opportunity to put in place a secondary prevention but the detection of these brain lesions can only be achieved so far by cerebrospinal fluid (CSF) evaluation or molecular amyloid and tau PET imaging. There is an urgent need to find out simple and easily accessible new biomarkers to set up an efficient screening in adult and aging population. Neuropathological and biochemical studies have revealed that abnormal accumulations of potentially toxic kinases are present in the brains of AD patients. Kinase activation leads to abnormal tau phosphorylation, amyloid production, apoptosis and neuroinflammation. Increased levels of these kinases are present in the CSF of mild cognitive impairment (MCI) and AD patients. Over the last years the search for abnormal kinase levels was performed in the blood of patients. Glycogen synthase kinase 3 (GSK 3), protein kinase R (PKR), mamalian target of rapamycin (mTOR), dual specificity tyrosine-phosphorylation-regulated kinase 1A (DIRK1A), c-Jun N-terminal kinase (JNK), protein 70 kD ribosomal protein S6 kinase (P70S6K), ERK2 and other kinase concentrations were evaluated and abnormal levels were found in many studies. For example, GSK3 levels are increased in MCI and AD patients. PKR levels are also augmented in peripheral blood mononuclear cells (PBMC) of AD patients. In the future, the assessment of several blood kinase levels in large cohorts of patients will be needed to confirm the usefulness of this test at an early phase of the disease.

## Introduction

Neuropathological lesions in Alzheimer’s disease (AD) include amyloid plaques made of Aβ peptides, neurofibrillary tangles composed of hyperphosphorylated tau proteins, amyloid angiopathy and synaptic and neuronal loss (Duyckaerts et al., 2009). Recent findings concerning the pathological evolution of AD have converged and proposed that abnormal biochemical modifications of the brain, including Aβ accumulation, could occur one or two decades before the first clinical sign, reflecting the presence of a long clinically silent period of the disease (Jack et al., 2018). The amyloid cascade hypothesis proposes that Aβ oligomers could be toxic for neurons and could induce progressive neuroinflammation and neurodegeneration (Selkoe and Hardy, 2016). In this model, the activation of kinases by Aβ in neurons and other brain cell types could induce tau phosphorylation and the triggering of detrimental cellular pathways leading to neuronal demise and microglial activation (Iqbal et al., 2009). The current diagnosis of AD is now clearly facilitated by molecular imaging biomarkers or cerebrospinal fluid (CSF) biomarkers (Lista et al., 2015). Previous works have revealed that many kinase levels are modified in the brain and CSF of AD patients including glycogen synthase kinase 3 (GSK3; Pei et al., 1997), cyclin dependent kinase 5 (CDK 5; Baumann et al., 1993), c-Jun N-terminal kinase (JNK; Gourmaud et al., 2015), mamalian target of rapamycin (mTOR; Lafay-Chebassier et al., 2005), proapoptotic kinase R (PKR) (Chang et al., 2002b), protein kinase C (PKC; Masliah et al., 1990). Many of these kinases can phosphorylate tau but they are also pro-apoptotic and can lead to neurodegeneration (Fielder et al., 2017). The rational for assessing kinase levels or activities in blood is linked to the recent findings revealing that plasma Aβ levels could be associated with CSF Aβ concentrations in AD patients (Janelidze et al., 2016; Hanon et al., 2018). The links between kinase levels in the brain and in the blood or in blood cells are not fully understood but since kinase concentrations are enhanced or decreased in AD brains and CSF, it was proposed by several teams that evaluating blood kinase levels in AD patients and controls could be worth trying as a new search for easily accessible biomarkers. During the last years several studies have been performed in AD patients, mild cognitive impairment (MCI) and controls, comparing blood concentrations or activities of specific kinases involved in brain lesions. The major goal of these studies was to determine if the results of blood kinase levels could be utilized in the future as possible biomarkers in AD. It seems clear that evaluating on a large scale, individuals with or without cognitive symptoms using molecular imaging or CSF biomarkers is not realistic due to the cost or the invasive nature of the approaches. The screening of persons with putative AD brain lesions using a blood test seems an attainable goal. The justification of this preventive attitude will be fully justified when an appropriate and efficient disease modifying treatment for AD will be found. Large-scaled proteomics of the human kinome have been already developed but so far never extensively applied to possible AD patients (Oppermann et al., 2009). The use of kinase as biomarkers has already largely been assessed in oncology and has served as a validation for target engagements in drug discovery (Yu et al., 2007). The purpose of this brief review is to describe the major current results performed in AD or MCI due to AD patients using blood-based kinase evaluations. All studies published so far have not included the results of sensitivity, specificity and individual predictive values. In addition many studies have used semi-quantitative methods to assess kinase levels. The findings cannot be referred as real biomarkers but could only reflect abnormal blood kinase metabolisms occurring in AD patients.

## Glycogen Synthase Kinase 3 (GSK 3)

Glycogen synthase kinase 3 (GSK 3) is widely expressed in many human tissues and is present in peripheral blood mononuclear cells (PBMC). GSK3 is expressed with two isoforms α and β. GSK3 is implicated in tau phosphorylation and in Aβ precursor protein (APP) processing linking the two pathological processes in AD (Hooper et al., 2008). In an initial clinical study, the levels of GSK3 α and β and their respective regulating phosphorylated epitopes (serine 9 and serine 21) have been assessed using western blots in PBMC from AD patients (*N* = 60), in MCI (*N* = 33) and from healthy aging individuals (*N* = 20; Hye et al., 2005). The findings revealed that total levels of GSK 3 α and β were increased in AD and MCI patients as compared to controls but the seine 9 phosphorylated epitope was not significantly different in the three groups. The authors concluded that measuring GSK3 PBMC levels could be useful as diagnostic biomarker in AD. Another report has mentioned different results in a cohort of patients suffering from AD, MCI, depression and in normal individuals (Marksteiner and Humpel, 2009). Using an ELISA method in PBMC, the authors revealed that GSK3-β levels were significantly decreased only in MCI patients and no differences were observed in other groups. It was proposed that both GSK3-β and PI3K, which regulates GSK3-β, could be similarly modulated in PBMC. In 2011, a new research also using western blots in PBMC has shown in a cohort of 20 AD, 25 Parkinson’s disease patients and 30 healthy controls that the concentrations of total GSK3 α and β and their respective phosphorylated epitopes were significantly increased in AD as compared to controls, confirming the first results (Armentero et al., 2011). Further studies are needed in exploratory and confirmatory cohorts to determine the usefulness of GSK3 as a possible blood biomarker in MCI and AD patients.

## Cyclin Dependent Kinase 5 (CDK5)

Cyclin dependent kinase 5 (CDK5) is a key enzyme controlling tau phosphorylation and has been implicated in the pathogenesis of AD (Cruz and Tsai, 2004). To the best of our knowledge, a recent survey of published data has not found any evaluation of cdk5 in PBMC or serum in MCI or AD patients. In contrast, the cdk5 gene polymorphism seems to be a risk factor for AD. A first report in 2009 has shown in a cohort of 549 AD patients and 5728 controls that the haplotype GG of the SNP rs2069442 of cdk5 gene increased the risk for AD in ApoE4 negative patients. In incident non-ApoE4 AD cases, the risk was augmented by 1.9-fold (Arias-Vásquez et al., 2008). This type of association was not observed in a Polish population of 257 AD patients and 80 controls concerning cdk5 polymorphisms and AD risk factor nor with several other blood biochemical parameters (Czapski et al., 2012). The evaluation of cdk5 levels and activities in PBMC of AD and MCI patients could be worth trying to decipher putative abnormal cdk5 levels.

## MAPK Kinases: c-Jun N-terminal Kinase (JNK) and p38 Kinase

JNK is a serine-threonine mitogen activated protein kinase (MAPK) coded by three genes, JNK1, JNK2 and JNK3. JNK1 and JNK2 isoforms are widely found in tissues, whereas JNK3 is mainly located in the brain (Davis, 2000). Activated JNK (pJNK) accumulations have been detected in AD brains and we have demonstrated that JNK3 levels were enhanced in AD CSF, and were linked to cognitive decline in patients (Gourmaud et al., 2015). JNK can induce enhanced Aβ production, through the phosphorylation of APP on threonine 668 (Thr668), leading to an exacerbated amyloïdogenic processing (Standen et al., 2009). P38 is also a MAPK kinase that has been implicated in neuroinflammation and tau phosphorylation and is the focus of new targeting therapies in this disease (Munoz and Ammit, 2010). A recent study has revealed that assessing total and activated JNK and p38 PBMC levels can lead to interesting results in AD (Wang et al., 2014). The authors found in a small cohort of 20 AD patients and 20 controls that PBMC levels of activated JNK and p38 were significantly increased in AD and correlated with the duration of evolution and MMSE scores. Patients with low MMSE scores had increased PBMC levels of both MAPK kinases. Another recent data has demonstrated that the EDTA plasma levels of two other MAPK kinases were associated with AD. The levels of MAPKAPK5 were positively associated with the cognitive tests assessed over a period of 10 years and the levels of MAP2K4 were negatively associated with the volume of the left entorhinal cortex (Kiddle et al., 2015). Further studies will be needed to confirm these results in large validated cohorts.

## Mamalian Target of Rapamycin (mTOR)

The kinase mTOR has also been implicated in the pathogenesis of AD (Pei and Hugon, 2008). mTOR is a serine/threonine kinase originating from two genes TOR1 and TOR2. Only mTORC1 interferes with rapamycin. mTOR can control cell growth, cell proliferation, protein synthesis and autophagy. Among the mTOR downstream targets controlling translation, the protein 70 kD ribosomal protein S6 kinase (p70S6K) has also been studied in AD. Previous studies of mTOR and p70S6K levels in AD brains have found contradictory results with either decreased concentrations (Lafay-Chebassier et al., 2005) or increased levels (Caccamo et al., 2010). We have carried out, using western blots, a research in a cohort of 32 AD patients and 33 controls assessing the levels of mTOR and p70S6k in PBMC (Paccalin et al., 2005, 2006a,b). mTOR levels were significantly reduced in AD patients as compared to controls and these levels correlated with cognitive scores of the free and cued recall tests (FCRT), the MMSE scores and the reverse digit span tests. Similar findings were detected for p70S6k levels which correlated with FCRT, reverse digit span test an oral denomination test. AD patients at an early stage of the disease had increased levels of these kinases and rather preserved neuropsychological scores. In addition, p70S6k levels also correlated with the results of emotional memory tests. Although it is difficult to extrapolate these results to neurons and brain, it is known that stressed neurons can perhaps modulate protein translation and increase the expression of the APP (Lesort et al., 1997). The links between kinase levels in brain and PBMC need to be compared in future researches. Nevertheless assessing the PBMC levels of these kinases might be a useful way to determine new screening biomarkers in patients with memory complaints.

## Eukariotic Initiation Factor Kinase 2 (PKR2)

PKR is a proapoptotic serine/threonine kinase activated by virus, inflammatory and toxic cellular signals and is responsible for translation initiation blockade via the phosphorylation of its downstream target, the eukaryotic initiation factor two α (eIf2α). In addition to triggering apoptosis, PKR is involved in innate immunity and the process of inflammation that are typical features observed in AD brains (Dabo and Meurs, 2012). Using immunohistochemistry, we have demonstrated in 2002 that the levels of PKR and eIf2α were increased in AD brains as compared to control individuals. PKR labeling was often but not always associated with neurofibrillary tangles and hyperphophorylated tau protein (Chang et al., 2002a, b). Later we have observed increased levels of PKR and phosphorylated PKR (pPKR) in the CSF of AD and MCI patients as compared to neurological controls (Mouton-Liger et al., 2012). CSF activated PKR levels were predictive of cognitive decline of the patients; high CSF concentrations of PKR were observed in rapidly declining patients over a 2-year period (Dumurgier et al., 2013). We have also assessed the levels of total PKR and activated PKR and eIf2α in PBMC from AD patients and control individuals. The results demonstrated significant increased levels of these two cellular signals in AD patients. These concentrations were correlated with MMSE scores, FCRT, and reverse digit span cognitive tests (Paccalin et al., 2006b). High levels of PKR and eIf2α were detected in the more severely affected AD patients. These findings associated with the previous ones suggest that mTOR levels in PBMC are higher at the onset on the clinical disease whereas PKR levels are higher over the course of the disease. How the levels of these biomarkers are during the silent and preclinical periods of AD remains to be explored in order to determine their usefulness as markers of the biological phase of the disease (Jack et al., 2018). Although the links between cerebral and PBMC levels of these kinases are not very well known, it is worthy to notice that the kinase mTOR favors cell growth whereas the kinase PKR is part of the cellular apoptotic process.

## Protein Kinase C (PKC)

The PKC family includes transmembrane serine/threonine kinases regulating signal transduction and encompasses in mammals 12 isoenzymes including PKC-α, PKC-δ, and PKC-ζ. PKC deficit has been implicated in the pathogenesis of AD (Malik et al., 2015). It has been postulated that a PKC deficiency could lead to memory loss, increased Aβ levels, enhanced phosphorylated tau and augmented neuroinflammation. There are two studies reporting results of blood PKC levels in AD patients. In 2006, a manuscript showed abnormal PKC conformation in red blood cells of affected patients (Janoshazi et al., 2006). Using a new and specific fluorescent probe called Fim-1, the authors demonstrated that PKC conformation is modified in AD patients (*N* = 33) and not in Parkinson’s patients (*N* = 15) as compared with healthy individuals (*N* = 25). This alteration was not linked to age or to duration of disease and the authors proposed that this test could be a screening method in patients with cognitive problems. In 2008 another report using flow cytometry analyzed three PKC isoforms PKC-α, PKC-δ, and PKC-ζ in T-lymphocytes extracted from PBMC and exposed to Aβ. AD patients (*N* = 40) and healthy controls (*N* = 40) were explored. The findings have shown that Aβ activated T-lymphocytes from AD patients revealed highly expressed cell subpopulation with phosphorylated PKC-δ and PKC- ζ. This result was not observed in PBMC from controls as well as in freshly purified non Aβ-exposed T-lymphocytes from both groups (Ciccocioppo et al., 2008). The assessment of PKC in PBMC or red blood cells could be performed in further research to validate this approach as a screening tool in patients with cognitive complaints.

## Other Kinases or Phosphatases

Dual specificity tyrosine-phosphorylation-regulated kinase 1A (DIRK1A) is able to phosphorylate many substrates and is implicated in tau phosphorylation via an interaction with GSK3β. A recent study has demonstrated that serum levels of DIRK1A were modified in AD. Using slot-blotting method in human serum to detect kinase relative expression, the results in 26 AD patients and 25 controls revealed a significant reduction of DIRK1A levels in AD patients as compared to controls. Serum levels correlated with CSF tau and phosphorylated tau concentrations (Janel et al., 2014).

Phosphatases have also been explored as putative AD biomarkers. For example, thiamine diphosphatase and thiamine monophosphatase are implicated in thiamine metabolism and their heparin plasma activities were recently measured in 45 AD patients and 38 control individuals. Significantly increased activities of both phosphatases were detected in AD patients as compared to controls suggesting that a disturbed thiamine diphosphate metabolism was present in AD patients (Pan et al., 2017).

G-protein-coupled receptor kinase-2 (GRK2) is largely involved in the regulation of the G-protein-coupled receptors at the cell membrane. GRK2 is increased in the brain of AD patients (Obrenovich et al., 2006). Using qPCR and western blots, GRK2 mRNA and protein levels were explored in blood lymphocytes of mild (MMSE: 18–24) and moderate to severe (MMSE < 18) AD patients. The findings revealed that GRK2 mRNA and protein levels were significantly increased in both groups of AD patients with a major augmentation in severely affected patients (Leosco et al., 2007).

## Multi-Target Kinase Therapies and Biomarkers

There is so far no treatment able to block or attenuate AD brain lesions in humans and new disease modifying drugs are currently evaluated in many clinical trials. Kinase inhibitors have been already tested in AD without current success. For example, the GSK3 inhibitor tideglusib was assessed but no significant clinical outcomes were obtained (Lovestone et al., 2015). We have seen in this review that many kinases present in human brains are involved in signaling pathways implicated in AD brain lesions such as Aβ metabolism, tau phosphorylation, neuronal apoptosis and neuroinflammation. A new therapeutic approach targeting several kinases was recently highlighted (Tell et al., 2016). A pharmacological intervention in AD using this method will need blood companion biomarkers readily available to evaluate drug targeting of these new kinase inhibitors. In the future, blood-based kinase biomarkers could be included in diagnostic and therapeutic evaluations in preclinical patients or MCI due to AD patients.

## Conclusion

We have seen that many results concerning blood-based kinase biomarkers in AD need new findings in lager confirmatory cohorts and in longitudinal follow-ups of patients. There are several limitations in the studies reported so far in AD. (1) Very few publications have reported the results in confirmatory cohorts with a large numbers of patients. (2) Most of the studies have focused the research on one or a few kinase levels or activities but large-scaled proteomics focalized on this type of proteins should be worth assessing in MCI, AD patients and controls. Phospho-peptides could also be assessed on a large scale. (3) The real signification of blood kinase changes described in this review is not so far perfectly elucidated. Further studies will be needed to assess if these modifications originate from a general disturbed kinase metabolism or from signals coming from the AD brain.

It is plausible to envisage in the future that several kinase levels could be simultaneously assessed in the blood (PBMC, RBC Proceed, plasma) of patients. The use of an algorithm incorporating this kinome analysis might be useful to screen MCI or pre-symptomatic patients and eventually to compare these results with those obtained in the CSF of patients as seen for PKR and JNK3. Finally specific blood-based kinase biomarkers could be incorporated as surrogate markers of target engagement in new clinical trial assessing kinase inhibitors.

## Author Contributions

JH, EC and CP made the review of the literature and wrote the manuscript. FM-L and JD performed some experiments. All authors read and corrected the manuscript.

## Conflict of Interest Statement

The authors declare that the research was conducted in the absence of any commercial or financial relationships that could be construed as a potential conflict of interest.
